# Cost-effectiveness of MRI for breast cancer screening in BRCA1/2 mutation carriers

**DOI:** 10.1186/1471-2407-13-339

**Published:** 2013-07-10

**Authors:** Reka Pataky, Linlea Armstrong, Stephen Chia, Andrew J Coldman, Charmaine Kim-Sing, Barbara McGillivray, Jenna Scott, Christine M Wilson, Stuart Peacock

**Affiliations:** 1 Cancer Control Research, BC Cancer Agency, 675 W. 10th Ave, Vancouver, BC V5Z 1L3, Canada; 2 Canadian Centre for Applied Research in Cancer Control, 675 W. 10th Ave, Vancouver, BC V5Z 1L3, Canada; 3 Provincial Medical Genetics Program, BC Women’s Hospital and Health Centre, 4500 Oak Street, Vancouver, BC, V6H 3N1, Canada; 4 Hereditary Cancer Program, BC Cancer Agency, 600 W. 10th Ave, Vancouver, BC V5Z 4E6, Canada; 5 Department of Medical Genetics, Faculty of Medicine, University of British Columbia, 2350 Health Science Mall, Vancouver, BC V6T 1Z3, Canada; 6 Medical Oncology, BC Cancer Agency, 600 W. 10th Ave, Vancouver, BC V5Z 4E6, Canada; 7 Department of Medicine, Faculty of Medicine, University of British Columbia, 2775 Laurel St, Vancouver, BC V5Z 1M9, Canada; 8 Population Oncology, BC Cancer Agency, 686 W. Broadway, Vancouver, BC V5Z 1G1, Canada; 9 Radiation Oncology, BC Cancer Agency, 600 W. 10th Ave, Vancouver, BC V5Z 4E6, Canada; 10 Department of Surgery, Faculty of Medicine, University of British Columbia, 910 W. 10th Ave, Vancouver, BC V5Z 4E3, Canada; 11 Screening Mammography Program of British Columbia, 686 W. Broadway, Vancouver, BC V5Z 1G1, Canada; 12 Diagnostic Imaging, BC Cancer Agency, 600 W. 10th Ave, Vancouver, BC V5Z 4E6, Canada; 13 Department of Radiology, Faculty of Medicine, University of British Columbia, 950 W. 10th Ave, Vancouver, BC V5Z 4E3, Canada; 14 School of Population and Public Health, University of British Columbia, 2206 East Mall, Vancouver, BC V6T 1Z3, Canada

**Keywords:** Breast cancer, BRCA, MRI, Cost-effectiveness, Screening

## Abstract

**Background:**

Women with mutations in BRCA1 or BRCA2 are at high risk of developing breast cancer and, in British Columbia, Canada, are offered screening with both magnetic resonance imaging (MRI) and mammography to facilitate early detection. MRI is more sensitive than mammography but is more costly and produces more false positive results. The purpose of this study was to calculate the cost-effectiveness of MRI screening for breast cancer in BRCA1/2 mutation carriers in a Canadian setting.

**Methods:**

We constructed a Markov model of annual MRI and mammography screening for BRCA1/2 carriers, using local data and published values. We calculated cost-effectiveness as cost per quality-adjusted life-year gained (QALY), and conducted one-way and probabilistic sensitivity analysis.

**Results:**

The incremental cost-effectiveness ratio (ICER) of annual mammography plus MRI screening, compared to annual mammography alone, was $50,900/QALY. After incorporating parameter uncertainty, MRI screening is expected to be a cost-effective option 86% of the time at a willingness-to-pay of $100,000/QALY, and 53% of the time at a willingness-to-pay of $50,000/QALY. The model is highly sensitive to the cost of MRI; as the cost is increased from $200 to $700 per scan, the ICER ranges from $37,100/QALY to $133,000/QALY.

**Conclusions:**

The cost-effectiveness of using MRI and mammography in combination to screen for breast cancer in BRCA1/2 mutation carriers is finely balanced. The sensitivity of the results to the cost of the MRI screen itself warrants consideration: in jurisdictions with higher MRI costs, screening may not be a cost-effective use of resources, but improving the efficiency of MRI screening will also improve cost-effectiveness.

## Background

Carriers of BRCA1 or BRCA2 (BRCA1/2) mutations are at particularly high risk of breast cancer, with a 45-65% cumulative risk by age 70 years [1,2]. In current practice at the British Columbia Cancer Agency (BCCA), women with a strong family history of breast and ovarian cancer who meet specific eligibility criteria [3] may be referred to the Hereditary Cancer Program to receive genetic counseling and testing for BRCA1/2 mutations. Women with a BRCA1/2 mutation may significantly reduce their risk of breast cancer by opting to undergo prophylactic bilateral mastectomy and/or bilateral oophorectomy [4-7], but many factors are involved in choosing risk-reducing surgery [8] and many women instead opt for early detection strategies, including regular screening with MRI and mammography [9]. Since 2003, the BCCA has operated a high-risk screening clinic, offering annual breast cancer screening with MRI and mammography to confirmed BRCA1/2 mutation carriers.

MRI is more sensitive than mammography for breast cancer screening in BRCA1/2 mutation carriers, with screening trials indicating that between 89-100% of breast cancers were detected with the combination of mammography and MRI, versus 33-50% with mammography alone [10-18]. However, the specificity of MRI is lower than mammography (73-80% for mammography and MRI vs. 91-99% for mammography alone [10-18]), giving rise to more false positive screens, which may increase costs and negatively impact quality of life for screening participants [19]. Breast MRI is more expensive than mammography, but there is little evidence available on the cost-effectiveness of MRI for breast cancer screening in Canada. Estimates from the United States of incremental cost per quality adjusted life year (QALY) for the addition of MRI to annual mammography screening range widely, from $55,420/QALY [20] and $69,125/QALY [21] for BRCA1 carriers, $130,695/QALY BRCA2 carriers [20], and $179,599/QALY for women with >15% lifetime risk [22] (all values USD). Cost-effectiveness ratios are particularly sensitive to the unit cost of an MRI screening test [21-23] and to the breast cancer risk in the population being screened [20,24]. In order to better understand the context of MRI screening at the BCCA, the investigators determined a local cost-effectiveness analysis was warranted. The objective of this study is to estimate the cost-effectiveness of annual mammography plus MRI screening for breast cancer in BRCA1/2 mutation carriers, as compared to screening with mammography alone, from the perspective of the British Columbia healthcare system, using local cost and outcomes data.

## Methods

### Model design

**Figure 1:**
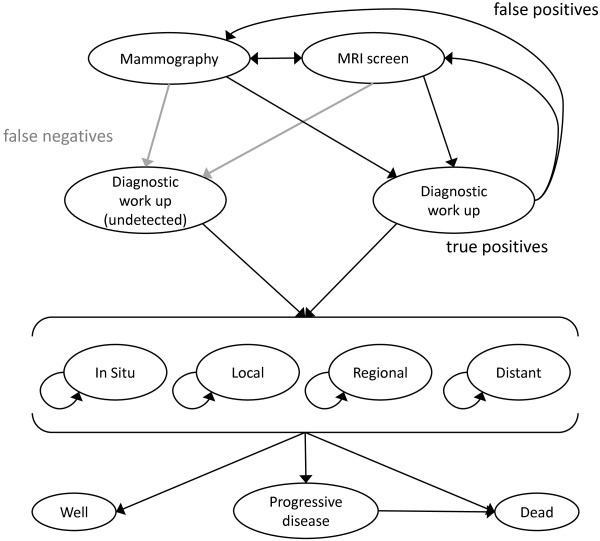
**Markov model for annual breast cancer screening with MRI and Mammography.** Women begin in the Markov stages “MRI screen” or “Mammography” and alternate every 6 months (in the mammography alone arm, the “MRI screen” state is replaced with a 6-month interval with no screen). Women with positive screening results move through the right side of the model; those with no cancer (false positives) return to screening, while cancer cases continue to treatment, by stage at diagnosis. Women with cancer whose screening results are negative (false negatives) are screened once more; if their cancer remains undetected they are classified as having non-screen-detected cancers, and proceed through treatment.

An advisory panel of clinicians, program managers and researchers was established to support this study. The investigators constructed a Markov model to determine the cost per QALY gained with current MRI and mammography screening practices, comparing annual mammography alone to annual mammography and MRI (Figure 1), from the perspective of the healthcare system. The model simulates a cohort of women beginning at age 25 years, with a 6-month cycle length, representing the current time between screens, and a lifetime time horizon. The model represents screening, diagnostics and treatment for a woman’s first breast cancer; screening for second primary cancers is not considered.

In the mammography plus MRI screening strategy, women alternate between MRI and mammography screening every six months (Figure 1). In current practice at the BCCA high-risk screening clinic, MRI is offered from ages 25–64 years, and mammography screening is offered from ages 30–79 years; thus in the mammography plus MRI strategy, women aged 25–29 years receive only MRI screening, and women 65–79 years receive only mammography. In the mammography alone strategy, women are screened with mammography annually from age 30–79 years. Women with cancer detected by screening (true positives) proceed through diagnostic work-up to treatment; women with false positive screen results also undergo a diagnostic work-up but return to screening. Any women with incident cancer that is not detected by screening remain in the screening health states for a further 6 months; if their cancer remains undetected (that is, if in the MRI arm their subsequent screen is also negative, or if in the mammography alone strategy they do not receive a screen within those 6 months), they are classified as having clinically manifesting non-screen-detected cancer.

Cancer treatments and outcomes by stage at diagnosis are the same across both strategies of the model. In the model, patients undergo treatment for the first 18 months following diagnosis, or until they die or transition to progressive disease, whichever is shorter. Patients who die of cancer within 18 months (3 cycles) of diagnosis transition to the ‘dead’ health state without moving through the ‘progressive disease’ state, while those who die in subsequent cycles are assumed to have experienced progressive disease for the last 18 months (3 cycles) prior to death [25]. In the model, patients with in situ disease do not progress to invasive disease, and all patients who survive at least 10 years after diagnosis are no longer at risk of progression.

### Transition probabilities

Age-specific breast cancer incidence in the BRCA1 and BRCA2 populations [2] was weighted to represent mutation frequency at the BCCA (59% BRCA1 and 41% BRCA2; Table 1). Sensitivity and specificity values for the mammography plus MRI arm were taken from a meta-analysis of MRI screening effectiveness studies [16]. The sensitivity of MRI or mammography given a prior false negative screen with the opposite modality was calculated using the reported sensitivity values for each screening modality alone and the sensitivity of detection by either MRI or mammography when both are offered together. Using these values, we were able to solve for the joint probability of detection by both MRI and mammography, and derive estimates for the conditional probability of detection by MRI given a false negative from mammography, and vice versa. For the mammography alone strategy, age-specific sensitivity and specificity values were used [26] to account for the early onset of breast cancer among BRCA1/2 carriers and the decreased sensitivity of mammography in younger women. The stage distributions of MRI-detected and mammogram-detected cancers in the BRCA1/2 population were synthesized using Dirichlet distributions for stage at diagnosis [11-13,27]. For clinically manifesting, non-screen-detected cancers, the historical stage distribution prior to screening was used [28]. In the model, stage distribution for screen-detected cancers was based only on method of detection, and was independent of prior screening.

**Table 1:** Model inputs for transition probabilities for cancer incidence, screen effectiveness, staging and survival

**Incidence**						
*10-year risk of breast cancer, by age *[2]	%	95% CI				
20-30	1.5	1.1-1.9				
30-40	8.6	6.9-11				
40-50	18	14-23				
50-60	20	17-25				
60-70	18	15-22				
**Screen effectiveness**	*Sensitivity*		*Specificity*			
	%	95% CI	%	95% CI		
MRI and Mammography [16]	94	90-97	77	75-80		
*MRI*	*77*	*70-84*	*86*	*81-92*		
*MRI given false negative mammogram*	*90*	87-93*				
*Mammography*	*39*	*37-41*	*95*	*93-97*		
*Mammography given false negative MRI*	*74*	68-80*				
*Mammography alone, by age*[26]						
30-40	63	42-85	89.4	88.6-90.2		
40-50	70	61-80	86.7	86.3-87.2		
50-60	81	73-89	87.3	86.8-87.9		
60-70	84	77-91	89.0	88.4-89.5		
**Stage distribution**	*MRI-detected*[11-13,27]*†*		*Mammography detected*[11-13,27]*†*		*Non-screen detected*[28]	
	%	95% CI	%	95% CI	%	95% CI
In Situ	16	10-22	27	17-38	5	3-6
Local	68	62-72	49	38-58	48	46-50
Regional	16	10-22	22	12-31	40	37-42
Distant	1	0-4	2	0-11	8	6-9
**Survival‡**	*5-yr*		*10-yr*			
	*(%)*	*95% CI*	*(%)*	*95% CI*		
In Situ	100	-	100	-		
Local	96.8	96.4-97.1	90.6	89.2-91.7		
Regional	88.8	84.7-91.6	71.2	63.1-76.9		
Distant	26.1	21.9-29.9	10.2	7.2-13.2		

* 95% CIs for conditional probabilities were estimated by sampling from beta distributions for sensitivity of MRI and mammography combined and sensitivity for each modality alone, and solving for the conditional probability.

† Calculated using cancers pooled from studies of MRI and mammography screening added to a uniform Dirichlet distribution.

‡ estimated from Weibull distribution parameters provided by BCCA Surveillance and Outcomes Unit for the general breast cancer population.

Local survival rates for the general breast cancer population were calculated using data from the BC Cancer Registry (including linked deaths data from the BC Vital Statistics Agency), and were fitted to a series of Weibull distributions [29] by the Surveillance and Outcomes Unit of the BCCA to generate the transition probabilities for the cancer outcomes in the model. The advisory panel validated this decision; the literature suggests that survival among BRCA1/2 carriers with breast cancer is no worse than for mutation-free controls [30]. Transition through the progressive disease state before death, described above, was implemented by introducing an 18-month lead time to the calculated survival curves. Published estimates of competing mortality in the BRCA1/2 population were also incorporated into the model [31].

### Costs

All costs included in the model are summarized in Table 2. The cost of mammography screening was estimated from the BC Medical Services Commission Fee Schedule for 2008 [32]. MRI screening cost was calculated as the mean of three cost estimates provided by the BCCA and two regional health authorities. The cost included radiologist, technologist and clerical staff costs, materials, support costs, and overhead. The cost of a diagnostic work-up was calculated as the weighted mean cost of consultations, diagnostic mammography, ultrasound, fine needle aspiration, core biopsy, and open biopsy delivered following abnormal screen results, using observed frequencies reported from the provincial screening program [33] and local unit cost estimates [32,34].

**Table 2:** Costs of screening, diagnostics and treatment

**Screening and diagnostics**	***Cost ($)***	***95% CI***
MRI screen	277	196-376
Bilateral mammogram	95	55-146
Diagnostic work-up	187	106-292
**Total treatment cost**	*Cost ($)*	95% CI*
In Situ	3,427	1,713-5,140
Local	10,940	1,997-27,335
Regional	23,612	5,075-56,124
Distant	15,645	4,171-34,561
Progression (end of life)	26,704	11,851-47,489

Abbreviations: *CI* Confidence interval.

* Patient-level total costs were fitted to gamma distributions to generate 95% CI.

We calculated treatment costs in the model using records from the BC Cancer Agency database (CAIS) for all breast cancer patients who underwent mutation testing at the Hereditary Cancer Program between 2002 and 2007 and were found to be BRCA1 or BRCA2 mutation carriers (n = 68). Surgery, radiotherapy and systemic therapy in the first 18 months following diagnosis were included in the cost calculation [32,35-37] and fitted to gamma distributions [29]. Costs were calculated separately for three 6-month intervals (from months 1–6, 7–12, and 13–18 following diagnosis) to correspond with model cycle length and ensure appropriate allocation of costs over time. Using the subset of patients who died of breast cancer before January 2009 (n = 10) we calculated the cost of radiotherapy and systemic therapy received in the last 18 months of life (as three 6-month intervals), and estimated costs of additional hospitalization, using published length of stay and per-diem costs [36,37].

### Utilities

Standard gamble utility weights obtained from the literature for breast cancer treatment by stage at diagnosis were applied for up to 18 months while patients were in the cancer treatment states (Table 3) [38]. After 18 months a remission utility was applied to all cancer stages, until transition to progressive disease or return to full health after 10 years. The screening and interval health states were assumed to have a utility value of 1.0. The utility for a diagnostic workup was derived from a published value for diagnostic mammography, and lasted for two weeks of the 6 month cycle [39]. Utilities for remission and diagnostic mammography, which had been measured using a visual analog scale, were scaled up to approximate standard gamble values [39,40].

**Table 3:** Health state utility weights

**Screening and diagnostics**		***95% CI***
MRI screening*	1.000	-
Mammography	1.000	-
Diagnostic workup	0.987	0.761-1.00
**Treatment†**		
In situ	0.965	0.463-1.00
Localized	0.860	0.330-1.00
Regional	0.675	0.315-0.929
Distant	0.380	0.211-0.564
Progression (end of life)	0.380	0.211-0.564
Remission	0.965	0.463-1.00
Dead	0.000	-
Well	1.000	-

Abbreviations: *CI* Confidence interval.

* Full utility (1.00) assumed for screening states and ‘Well’ state; not varied in probabilistic sensitivity analysis.

† Treatment utilities by stage at diagnosis applied for 18 months, after which ‘Remission’ value applied until progression and death or transition to ‘Well,’ 10-yrs post-diagnosis; utilities for localized, regional, distant disease, and disease progression states were derived from Schleinitz *et al.*[38], and utility for diagnostic work-up, remission and in situ disease were from Bonomi *et al.*[39].

### Analysis

The model was analyzed using TreeAge Pro 2012, 1.3.0. The model design was clinically validated by members of the advisory panel, and model estimates of incidence and mortality were verified against published values. We conducted a cost-effectiveness analysis to calculate the incremental cost effectiveness ratio (ICER) of screening with MRI, expressed as 2008 CAD$ per quality-adjusted life year (QALY). Costs and utilities were discounted at 3.5% per year [41]. Probabilistic sensitivity analysis was conducted using Monte Carlo simulation techniques with 10,000 draws from the input distributions. Decision uncertainty was represented by plotting all results on the cost-effectiveness plane and by using the cost effectiveness acceptability curve, which illustrates the probability that MRI screening is cost-effective for a given range of willingness to pay values [29]. Cost-effectiveness was evaluated at example willingness to pay values of $50,000 and $100,000 per QALY. One-way sensitivity analysis was also conducted for the cost of MRI, sensitivity and specificity of MRI, stage of MRI-detected cancers, and discount rate to evaluate their impact on the ICER.

Ethical approval for this study was provided by the University of British Columbia-BC Cancer Agency Research Ethics Board.

## Results

After modeling a BRCA1/2 cohort from age 25 years, the cumulative risk of developing breast cancer by age 65 years was 42.7% (95% CI: 38.8, 46.7) (Table 4). This was slightly lower but generally comparable to cumulative incidence estimated from Antoniou *et al.*[1] and Chen and Parmigiani [2] (50.4% and 45.4% respectively), and considered a valid approximation. Mortality was slightly reduced with the addition of MRI, with 80.1% (95% CI: 78.9, 81.1) vs. 79.1% (95% CI: 77.4, 80.4) of women surviving to age 65. Mortality approximated values from Byrd *et al*. [42]; using data from that study, an estimated 22% of female BRCA1/2 mutation carries died before age 65 years from breast cancer or other causes, excluding ovarian cancer.

**Table 4:** Cost-effectiveness and effectiveness of MRI screening vs. annual mammography alone

Cost-effectiveness*	***Mammography only***		***MRI *&*mammography***		***Increment***	
	*Mean*	*95% CI*	*Mean*	*95% CI*	*Mean*	*95% CI*
Cost ($)	5,201	3,303-7,911	9,893	7,459-12,942	4,692	3,084-6,504
QALYs	22.57	21.86-22.75	22.66	21.85-22.83	0.092	−0.027-0.190
C/E ($/QALY)	231	146-351	437	329-572	50,911	Dominated-213,794
**Screening effectiveness†**	*Mammography only*		*MRI* &*mammography*			
	*Mean (%)*	*95% CI*	*Mean (%)*	*95% CI*		
Incidence	42.7	38.8-46.7	42.7	38.7-46.7		
Program sensitivity‡	71.7	65.9-77.2	93.9	89.4-97.3		
Stage distribution						
In Situ	20.8	12.8-30.1	18.2	12.8-24.2		
Local	48.7	39.1-58.3	61	53.8-67.8		
Regional	26.9	19.6-35.5	19.1	13.9-25.1		
Distant	3.6	2-7.2	1.7	0.6-3.8		
Survival	79.1	77.4-80.4	80.1	78.9-81.1		

Abbreviations: *MRI* magnetic resonance imaging, *QALY* quality-adjusted life year.

* Costs and utilities discounted at 3.5% per year.

† Calculated at end of MRI plus mammography screening program (age 65 years).

‡ Proportion of cancers in model detected by screening; all screen-detected cancers (true positives) divided by all incident cancers.

With the addition of MRI to annual mammography screening, 93.9% (95% CI: 89.4, 97.3) of cancers that developed by age 65 years were screen-detected, compared to 71.7% (95% CI: 65.9, 77.2) with mammography alone. Cancers in the MRI plus mammography arm were less likely to be either regional or distant, and more likely to be localized than in the mammography alone arm.

**Figure 2:**
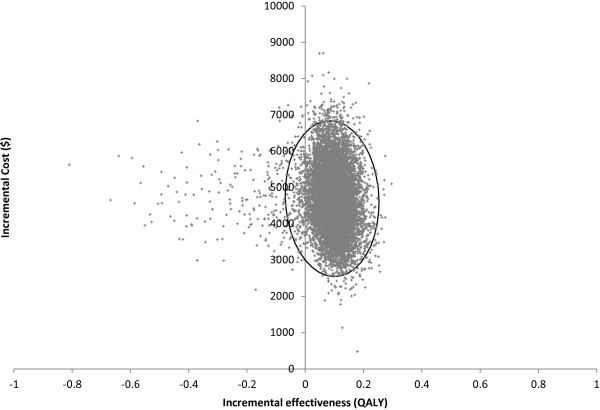
**Scatter plot of incremental cost and effectiveness for 10,000 simulations.** Ellipse indicates 95% confidence limit. Abbreviations: MRI, magnetic resonance imaging; QALY, quality-adjusted life-year.

**Figure 3:**
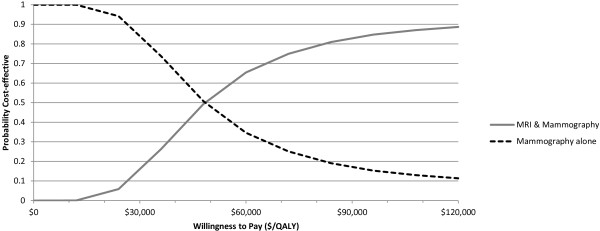
**Cost effectiveness acceptability curve.** Abbreviations: MRI, magnetic resonance imaging; QALY, quality-adjusted life-year.

Cost-effectiveness analysis gave an incremental cost of MRI screening of $4692 (95% CI: 3084, 7910) per participant, with 0.092 QALYs gained (95% CI: -0.027, 0.190), resulting in a mean ICER of $50,911/QALY (Table 4). The scatter plot of incremental cost and effectiveness values for each simulation is shown in Figure 2; in 3.9% of simulations, the MRI screening strategy was less effective than mammography alone. The cost-effectiveness acceptability curve (Figure 3) indicates that if a decision maker were willing to pay $100,000 per QALY gained, MRI screening is a cost-effective option 85.6% of the time. At a willingness to pay of $50,000/QALY, it is cost-effective 52.6% of the time.

**Figure 4:**
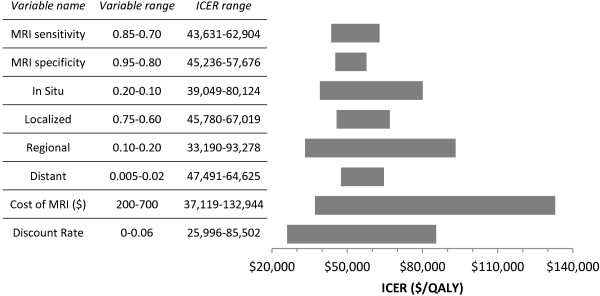
**One-way sensitivity analysis and tornado diagram of incremental cost**-**effectiveness of MRI screening.** Abbreviations: MRI, magnetic resonance imaging; ICER, incremental cost-effectiveness ratio; QALY, quality-adjusted life year.

One-way sensitivity analysis indicated that the model was somewhat sensitive to changes in the effectiveness of MRI screening – measured as MRI sensitivity, MRI specificity and stage distribution of MRI-detected cancers – but very sensitive to the cost of an MRI scan (Figure 4). As cost was varied from $200-$700, the ICER ranged widely, from $37,119 to $132,944 per QALY.

## Discussion

In our model, annual mammography plus MRI, compared to annual mammography alone, has an ICER of $50,900 per QALY. This ICER was estimated using local cost and treatment data, with input from clinicians and decision-makers on the project’s advisory panel, in an effort to most accurately depict the context of breast cancer screening and treatment for BRCA1/2 mutation carriers in British Columbia. These results suggest that the cost-effectiveness of the MRI screening program for BRCA1/2 mutation carriers is finely balanced, with sensitivity to input parameters and statistical uncertainty. The BCCA does not use a cost-effectiveness threshold, but the ICER falls within the generally accepted range for funded programs.

The mammography plus MRI strategy of the model differs from the mammography alone strategy in four key ways: the cost of MRI screening, increased screening sensitivity, a more favourable stage distribution among MRI-detected cancers, and more false positive screens due to decreased screening specificity. The cost-effectiveness of MRI screening is highly dependent on the cost of an MRI scan, as indicated in one-way sensitivity analysis. In situations where MRI scans are costlier than at the BCCA, MRI screening for breast cancer may not be a cost-effective option. However, these results also suggest that improvements in technical efficiency leading to reductions in the per-scan cost of MRI may reduce the cost-effectiveness of MRI screening to more acceptable levels.

The time horizon of this model, as with any model of preventive or screening techniques, also has an impact on the findings. The costs of MRI screening accrue from the beginning of model, while the benefits arising from MRI screening, such as lower treatment costs for cancers detected at an earlier stage, appear much later in the model, particularly as the cohort ages and cancer incidence rises. Consequently, the model is very sensitive to discounting assumptions for cost and QALYs.

The ICER calculated in this study is higher than previously published cost-effectiveness estimates from the UK, but lower than those from the US [20-22,24]. In the UK study by Norman *et al.*, women were screened for only 10 years, beginning at age 30 or 40 years, giving ICERS of approximately CAD$17,600 and $30,600 per QALY [24]. By contrast, our model includes MRI screening from age 25 to 65 years. In the US studies, the cost of MRI was much higher than in this study, around USD$1000 for a bilateral screen, which is a potential reason why the reported ICERs are also higher. Moore *et al.* found in their sensitivity analysis that reducing the cost of MRI to below USD$315 resulted in an ICER of under USD$50,000/QALY, down from the base-case ICER of nearly USD$180,000/QALY, which is more consistent with the findings of this study [22]. Both Moore’s model and this study highlight the fact that MRI screening for breast cancer may be cost effective, when the cost of MRI scans is low.

A limitation of this model is that it represents an idealized screening program, with all women entering at age 25 and participating until age 65 or until they develop cancer. The MRI screening program operated by the BCCA has a dynamic population. Women join the program at various ages when they are deemed to be eligible, and leave after undergoing prophylactic surgery, after developing cancer, or for other reasons. The timing of screening also varies: women who must travel to Vancouver for screening often have both MRI and mammography done concurrently, and the interval between MRI screens may exceed 12 months. Consequently, the cost-effectiveness of the screening program, if it were to be measured using real-world, comparative effectiveness program data, may be different. Although our goal was to use as much local data as possible, the challenge of acquiring comparative effectiveness data to inform the model was a further limitation of this study. We had insufficient sample size and follow-up to fully evaluate the effectiveness of the BCCA’s MRI screening program. We instead relied on the literature for screening effectiveness data. A further limitation of the model is that we were unable to include the risk of overdiagnosis from additional screening with MRI. Estimates of overdiagnosis attributable to mammography screening vary widely, from under 10% to as high as 50% [43-46]; however, overdiagnosis from MRI screening has not been assessed, nor has the rate of overdiagnosis in the BRCA1/2 population.

The model that we constructed to assess the cost-effectiveness of MRI screening lays the foundation to potentially address other questions related to breast cancer screening. For example, as more data become available the model could be adapted to find the optimal start time and duration of MRI screening from a cost-effectiveness perspective, or to investigate the relationship between lifetime breast cancer risk and cost-effectiveness of MRI screening, exploring the feasibility of expanding MRI screening to other high-risk groups.

## Conclusions

Annual mammography plus MRI screening of BRCA1/2 mutation carriers at the BCCA was found to be potentially cost-effective, with an ICER of $50,900/QALY when compared to annual mammography alone, although the cost-effectiveness is finely balanced. The benefits of early detection of breast cancer with MRI in this population may outweigh the added cost of screening and the higher risk of false positives; however, the cost-effectiveness of MRI screening is highly dependent on the cost of MRI scans and there remains some statistical uncertainty around the results.

## Abbreviations

BC: British Columbia; BCCA: BC Cancer agency; CI: Confidence interval; ICER: Incremental cost-effectiveness ratio; MRI: Magnetic resonance imaging; QALY: Quality-adjusted life-year.

## Competing interests

The authors declare that they have no competing interests.

## Authors’ contributions

All authors contributed significantly to the model design and validation, interpretation of results, and to critical review of this manuscript. RP was responsible for model construction, data analysis and composing the manuscript. JS, CW, CKS, and AC provided data. All authors read and approved the final manuscript.

## Pre-publication history

The pre-publication history for this paper can be accessed here:

http://www.biomedcentral.com/1471-2407/13/339/prepub
